# Associations between unpasteurised camel and other milk consumption, livestock ownership, and self-reported febrile and gastrointestinal symptoms among semi-pastoralists and pastoralists in the Somali Region of Ethiopia

**DOI:** 10.1017/S0950268822000450

**Published:** 2022-05-02

**Authors:** A. A. Roess, F. M. Hosh, L. C. Morton, N. Bestul, J. Davis, L. Carruth

**Affiliations:** 1 College of Health and Human Services, George Mason University, Fairfax, Virginia, USA; 2 Milken Institute School of Public Health, George Washington University, Washington, DC, USA; 3 Public Health Consultant, Dire Dawa, Ethiopia; 4 School of International Service, American University, Washington, DC, USA

**Keywords:** unpasteurized camel milk, Ethiopia, infectious disease symptoms

## Abstract

Contact with livestock and consumption of unpasteurised dairy products are associated with an increased risk of zoonotic and foodborne infection, particularly among populations with close animal contact, including pastoralists and semi-pastoralists. However, there are limited data on disease risk factors among pastoralists and other populations where livestock herding, particularly of dromedary camels, is common. This cross-sectional study used a previously validated survey instrument to identify risk factors for self-reported symptoms. Adults (*n* = 304) were randomly selected from households (*n* = 171) in the Somali Region of Ethiopia, a region characterised by chronic food insecurity, population displacement, recurrent droughts and large semi-pastoralist and pastoralist populations. Multivariable logistic regression assessed associations between self-reported symptoms and type of milk consumed, controlling for demographics and human-animal interaction. Consumption of days-old unrefrigerated raw camel milk was significantly associated with symptoms in the 30 days prior to the survey (AOR = 5.07; 95% CI 2.41–10.66), after controlling for age, refugee status, sanitation, camel ownership and source of drinking water and accounting for clustering. Consumption of days-old unrefrigerated raw ruminant milk was significantly associated with symptoms (AOR = 4.00, 95% CI 1.27–12.58). Source of drinking water and camel ownership, a proxy for camel contact, were significantly associated with the outcome in each model. There were no significant associations between self-reported symptoms and fresh or soured animal milk consumption. Research is needed to identify pathogens and major routes of transmission. Tailored communication campaigns to encourage safe food preparation should also be considered.

## Introduction

Consumption of livestock products including raw or unpasteurised dairy products are primary sources of nutrition in many rural areas in low- and middle-income countries, particularly among pastoralists and semi-pastoralist populations in Africa [1–5]. Throughout the world, including in much of Africa and the Middle East, and in many high-income countries, consumption of raw dairy products is increasing and is frequently believed to be a ‘health food’ with benefits to digestion, immunity and the gut microbiome [6]. However, there are several known risks of consuming raw or unpasteurised dairy products [6, 7], especially where refrigeration and/or pasteurisation are lacking.

Pathogens such as *Campylobacter*, *Brucella, Salmonella* and *Mycobacterium bovis* can be easily transmitted via direct contact with livestock and consumption of unpasteurised milk and milk products [8–13] yet there remains few interventions to reduce this risk [14]. For example, Brucellosis is caused by *Brucella spp.* bacteria – a notable potential biothreat agent – and can be spread through both close contact with camels and consumption of raw camel milk [15]. Additionally, *Mycobacterium bovis* can also cause TB disease in people [16]. The high rates of tuberculosis in parts of East Africa where HIV rates are low and exposure to livestock is high suggests the need at least explore the possibility of bovine TB in livestock or raw livestock milk [8–10, 14].

Dromedary camels are central to the economies and cultures of millions of people in Ethiopia and in East and North Africa in general, including ethnic Somalis residing throughout the Horn of Africa [17]. Many households raise small herds of camels and other livestock outside regulatory systems, and without access to high-quality veterinary care and vaccinations [3, 5]. Camels in the Horn of Africa are raised for foods including fresh milk, soured milk, yoghurts and meat, as well as for secondary products such as hides and hair; camels are thus a major source of food, household materials, income and savings [17–19]. Additionally, camels and camel products are an important export commodity, especially to markets in the Middle East [20].

Unpasteurised soured and fresh livestock milk, including camel milk, are central to Somalis' diets [3, 4, 17]. Populations are therefore at risk of foodborne illnesses transmitted through the consumption of these milk and other food products. However, little research has been done to investigate the potential sources and risk factors of zoonotic and foodborne infectious diseases among pastoralist populations potentially at high risk [7]. Although zoonotic and foodborne disease interventions in domesticated, non-mobile livestock have proven successful, surveillance and control for camels and other livestock herded by nomadic and semi-nomadic pastoralists are challenging because of these populations' mobility, the informality of these livestock economies, and the poverty and disintegration of many local and regional agricultural and health bureaus in Ethiopia [3, 4, 21]. Understanding the potential risks of human exposures to zoonotic and foodborne pathogens in livestock and livestock milk is thus central to promoting food safety, food security and public health. To begin to fill gaps in knowledge about the unique risks pastoralists and Somalis face, this study was examined the associations between self-reported emerging zoonotic and foodborne illness and human-animal interaction, and food animal preparation and consumption behaviours.

## Methods

### Setting

The Somali Region of Ethiopia is located in one part of a partitioned territorial expanse of East Africa populated and governed primarily by ethnic Somalis, spanning Djibouti, the eastern third of Ethiopia, the Northern Frontier District of Kenya, Somaliland and Somalia. Somalis represent a minority ethnic group within the Federal Democratic Republic of Ethiopia and reside mostly in the arid, eastern third of the country [22–24]. The region is home to a large pastoralist population where livestock herding, particularly of dromedary camels, is a common occupation [21]. This region is also home to many resettled refugees and internally displaced persons who are vulnerable to food insecurity [17, 18, 21, 23]. Infant and maternal mortality rates in the Somali Region are higher than elsewhere in the country; and access to and use of regulated primary healthcare facilities is lower than in other regions [22, 23, 25].

### Study population

**Fig. 1.**
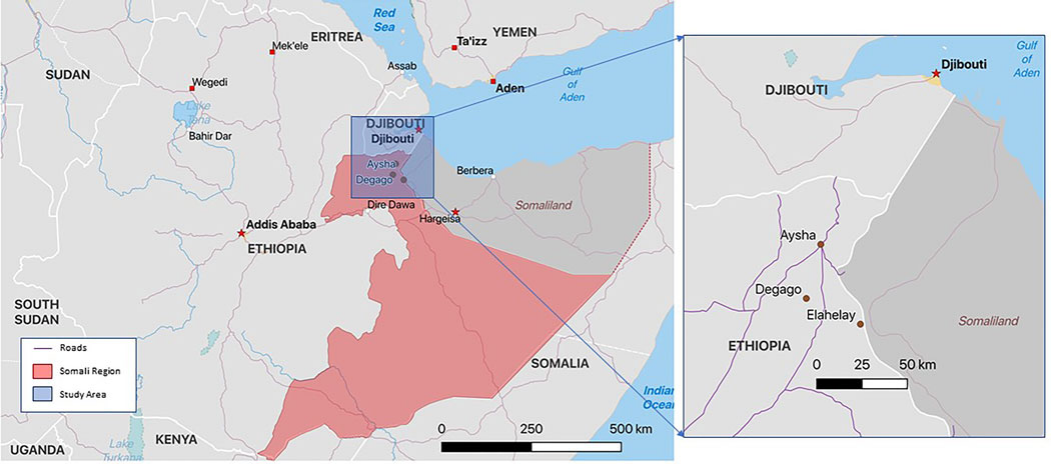
Map of Aysha, Degago and Elahelay in Somali Region, Ethiopia.

This study was conducted from September 2016 to May 2017 in Aysha District (a district is referred to as a *Woreda*) where one of the authors (L.C.) has conducted ethnographic research since 2007 [7, 17, 21, 23, 26]. Three community (called *kebele*) field sites (Degago, Elahelay and Aysha) located within the larger Aysha District were purposively selected based on the following criteria: prevalence of livestock and/or livestock products, transit of livestock through the area during at least one season of the year, low levels of political insecurity and violence, and different distances to a major road (see Fig. 1).

**Fig. 2:**
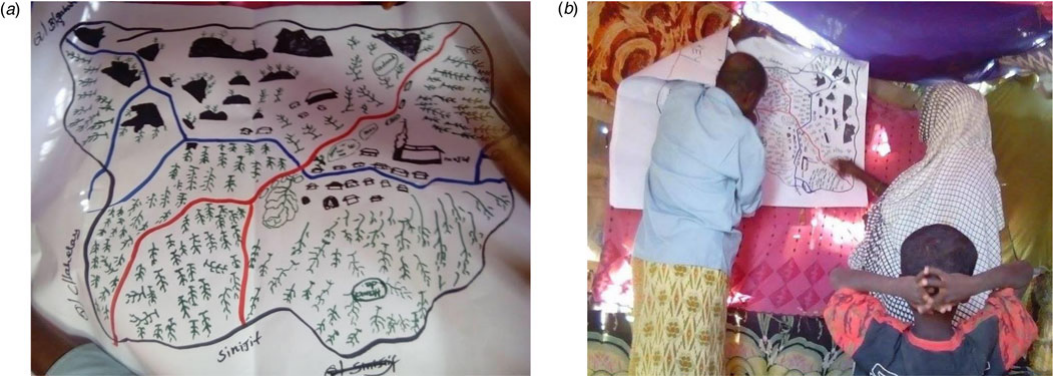
(a) Hand drawn map following participatory mapping exercise (b) Participatory mapping exercise

Two participatory mapping exercises were conducted in each community – one led by women with women, and one led by men with men, since men and women both have contact with and own livestock, but do so in different ways, and for most daily activities, are typically segregated by gender [27, 28]. Maps were hand drawn following the participatory mapping exercise to show where animals live and move in relation to houses and other structures (markets, clinics, mosques, etc.) (see Fig. 2). GPS coordinates were then collected for all of the household and other structures in the kebeles. Adults over the age of 14 years (the approximate age in this location when people can assume many adult responsibilities, such as marriage and/or care for livestock) who lived in the study site and were interested in participating in the study were eligible to participate. Households were randomly selected and 304 adults over the age of 14 years completed the survey. Every 5th household was selected from the list of households that was then generated. When possible one male and one female from each household were interviewed.

### Survey design

This was a cross-sectional study using an interviewer-led validated survey instrument to collect demographic data, self-reported symptoms, health care-seeking, animal husbandry, food animal preparation and consumption practices and sanitation variables. The interviews were conducted by local health workers from the Aysha District (*Woreda*). Data were entered using REDCap electronic data capture tools [29], and data analysis was performed using Stata version 16.1 (StatCorp, College Station, TX).

### Sample size

A sample size of 300 was sufficient to detect an association between self-reported symptoms and camel milk consumption, assuming that 25% of respondents with an exposure (consumption of soured camel milk) report a symptom, and 10% of those reporting no exposure also report no symptoms (*α* = 0.05, *β* = 0.2, Power = 0.8).

### Data analysis

Descriptive analysis of independent variables assessed the distribution of the data. Chi-squared and exact tests assessed the associations between self-reported illness (the outcome), type of milk consumed (the main exposure variable), and independent variables including demographic, livestock ownership (a proxy for animal contact), and sanitation variables. Variables associated with the outcome at a *P*-value of 0.05 or less and variables reported in the literature to be important were included in the final multivariable logistic regression models.

Multivariable logistic regression assessed the association between self-reported illness and the type of milk consumed while adjusting for potential confounders and household level clustering [30].

### Ethical review

Informed consent for interviews was obtained from all respondents. The protocols and consent forms used for this study were reviewed and approved by both the American University Institutional Review Board (IRB-2017-7) and Jigjiga University in Ethiopia.

## Results

A total of 304 individuals from 171 households participated in the study. The average age of participants was 44 years, with slightly more females interviewed (151, 49.7%) than males (124, 40.8%) (Table 1). Nearly all of the study participants reported being a member of the Issa *(Ciise)* Somali family group or ‘clan’ (272, 89.5%) and all spoke Somali *(Af-Soomaali)* as their first language. The majority reported at some time being a refugee (126, 41.5%) or an internally displaced person (106, 34.9%). Nearly all reported that they were recipients of the Productive Safety Net Programme (265, 87.2%), an assistance programme supported by the Government of Ethiopia and the United Nations World Food Program to increase the long-term resilience of chronically food-insecure households. Very few households had access to either electricity in their home or compound (43, 14.1%) or piped water inside of their dwelling (7, 2.3%), despite the ongoing local governmental efforts to provide these services, and nearly a third reported open defecation (86, 28.3%).

**Table 1.** Characteristics of the study population and unadjusted odds ratios (UOR) for self-reported symptoms – Somali Region, Ethiopia 2016–2017 (*n* = 304)

Variable	Total *n*	Per cent^a^	n (%) No symptoms	*n* (%) Symptoms	UOR (95% CI)
Age					
15–29	34	11.2	25 (73.5)	9 (26.5)	1
30–44	141	46.4	103 (73.1)	38 (27.0)	1.02 (0.44–2.39)
45–59	69	22.7	53 (76.8)	16 (23.2)	0.84 (0.33–2.16)
60+	46	15.1	38 (82.6)	8 (17.4)	0.58 (0.20–1.72)
Gender					
Female	151	49.7	117 (77.5)	34 (22.5)	1
Male	124	40.8	93 (75.0)	31 (25.0)	0.87 (0.50–1.52)
Refugee					
No	156	51.3	123 (78.9)	33 (21.2)	1
Yes	126	41.5	90 (71.4)	36 (28.6)	1.49 (0.86–2.57)
Displaced person					
No	184	60.5	149 (81.0)	35 (19.0)	1
Yes	106	34.9	70 (66.0)	36 (34.0)	0.98 (0.92–1.05)
Recipient of productive safety net programme					
No	21	6.9	14 (66.7)	7 (33.3)	1
Yes	265	87.2	202 (76.2)	63 (23.8)	0.62 (0.24–1.61)
Electricity					
No	221	72.7	169 (76.5)	52 (23.5)	1
Yes	43	14.1	33 (76.7)	10 (23.3)	0.98 (0.45–2.13)
Drinking water source					
Piped water inside of house	141	46.4	137 (84.1)	26 (16.0)	1
Well or other source outside of compound	163	53.6	96 (68.1)	45 (31.9)	2.47 (1.43–4.28)
Toilet					
Latrine or open defecation	127	41.8	88 (69.3)	39 (30.7)	1
Toilet	177	58.2	145 (81.9)	32 (18.1)	0.50 (0.29–0.85)

a Percentages do not add up to 100% due to missing data.

Among those reporting their primary occupation, the most common was agricultural (e.g. livestock herding or farming) (148, 47.4%), trading (44, 14.5%), and unemployed or unable to work (47, 15.5%) (Table 1). We found no association between self-reported symptoms and access to electricity, receiving Productive Safety Net Programme aid, or being displaced. There was a significant association between symptoms and having a source of drinking water that was outside of the home and between used a latrine or open defecation (Table 1).

More people owned small ruminants (87.8%) than camels (30.3%) and cows (4.0%) and only camel ownership was associated with a significant odds of self-reported symptoms (OR 6.00; 95% CI 3.27–10.99) (Table 2). Consumption of animal milk was common; 84.9% reported consumption of small ruminant milk, 66.1% reported consumption of camel milk, and 41.1% of cattle milk (Table 2).

**Table 2.** Prevalence of animal ownership and milk consumption behaviours and unadjusted odds ratio (UOR) with self-reported symptoms – Somali Region, Ethiopia 2016–2017 (*n* = 304)

Variable^a^	*n*	Per cent	*n* (%) No symptoms	*n* (%) Symptoms	UOR^b^	95% CI
**Livestock ownership**^c^						
**Goats/sheep**	267	87.8	206 (77.2)	61 (22.9)	0.44	0.17–1.14
**Camels**	92	30.3	50 (54.4)	42 (45.7)	6.00***	3.27–10.99
**Cattle**	12	4.0	9 (75.0)	3 (25.0)	1.06	0.28–4.03
**Milk consumption**^d^						
**Goats/Sheep**	258	84.9	196 (76.0)	62 (24.0)	1.30	0.59–2.84
**Camels**	201	66.1	145 (72.1)	56 (27.9)	2.27**	1.21–4.25
**Cattle**	125	41.1	108 (86.4)	17 (13.6)	0.36***	0.20–0.67
**Fresh milk consumption**^**d**^						
**Goats/Sheep**	242	79.6	187 (77.3)	55 (22.7)	0.85	0.44–1.61
**Camels**	216	71.1	164 (75.9)	52 (24.1)	1.15	0.63–2.09
**Cattle**	135	44.4	115 (85.2)	20 (14.8)	0.40**	0.23–0.72
**Not soured, old milk consumption**^**d**^						
**Goat/sheep**	30	9.9	12 (40.0)	18 (60.0)	6.25***	2.84–13.78
**Camel**	47	15.5	25 (53.2)	22 (46.8)	3.74***	1.95–7.17
**Cow**^e^	5	1.6	5 (100.)	0	–	–
**Soured milk consumption**^**d**^						
**Goat/sheep**	92	30.3	57 (62.0)	35 (38.0)	3.00***	1.73–5.22
**Camel**	73	24.0	41 (56.2)	32 (43.8)	3.84***	2.16–6.84
**Cow^e^**	6	2.0	6 (100.0)	0	–	–

a Variable categories are not mutually exclusive, participants were to select all that apply.

b Unadjusted odds ratios for self-reported symptoms (fever, vomiting and diarrhoea within the past 30 days).

c Reference group is not owning the specified livestock.

d Reference group is not consuming the specified type of milk.

e Unadjusted odds ratios not calculated due to small sample size.

**P < 0.01, ***P < 0.001.

Seventy-one people (23.4%) reported fever, diarrhoea and/or vomiting in the 30 days prior to the survey and 17 (5.6%) reported having been diagnosed with TB by a health care worker. There was no association between TB diagnosis and self-reported fever, diarrhoea and/or vomiting (data not shown). There was a significant positive association between self-reported symptoms within the past month and camel milk consumption (OR 2.27, 95% CI 1.21–4.25) but a negative association between symptoms and cattle milk consumption (Table 2). Consumption of soured camel and goat/sheep milk was associated with an increased odds of symptoms (OR 3.84, 95% CI 2.16–6.84 and OR 3.00, 95% CI 1.73–5.22, respectively) in the bivariable analysis (Table 2).

A multivariable logistic regression was constructed for each type of milk consumed.

In each model, camel ownership was significantly associated with an increased odds of self-reported illness after controlling for type of milk consumed, age, refugee status, use of toilets and source of drinking water (Table 3). After controlling for camel ownership, age, refugee status, use of toilets and source of drinking water, consumption of old camel milk and of old small ruminant milk remained significantly associated with an increased odds of symptoms (AOR = 2.51, 95% CI 1.15–5.49 and AOR = 4.00, 95% CI 1.27–12.58, respectively) (Table 3). Source of drinking water outside of the home was significantly associated with an increased odds of self-reported symptoms in each model (Table 3).

**Table 3.** Adjusted Odds Ratios (AOR) for self-reported symptoms (outcome) after controlling for demographics for each type of milk consumed

	Model 1: fresh camel	Model 2: old camel	Model 3: soured camel	Model 4: fresh small ruminant	Model 5: old small ruminant	Model 6: soured small ruminant
Milk consumption	0.77 (0.35–1.68)	2.51 (1.15–5.49)*	2.06 (0.99–4.28)	0.99 (0.42–2.34)	4.00 (1.27–12.58)**	2.00 (0.98–4.10)
Camel ownership	6.36 (3.12–12.96)***	5.07 (2.41–10.66)***	5.15 (2.48–10.69)***	6.16 (3.09–12.30)***	5.92 (2.92–12.01)***	5.57 (2.72–11.39)***
Age	0.99 (0.97–1.01)	0.99 (0.97–1.01)	0.99 (0.97–1.01)	0.99 (0.97–1.01)	0.99 (0.97–1.01)	0.98 (0.97–1.01)
Refugee status	1.77 (0.87–3.59)	1.90 (0.91–3.95)	1.80 (0.89–3.67)	1.75 (0.86–3.55)	1.94 (0.93–4.03)	1.72 (0.85–3.49)
Toilet	0.70 (0.35–1.42)	0.75 (0.37–1.52)	0.77 (0.38–1.58)	0.67 (0.33–1.34)	0.83 (0.39–1.74)	0.82 (0.40–1.67)
Drinking water	3.03 (1.49–6.17)**	2.61 (1.27–5.34)**	2.84 (1.39–5.82)**	2.94 (1.43–6.02)**	2.44 (1.20–4.99)**	3.00 (1.47–6.13)**

*P < 0.05, **P < 0.01, ***P < 0.001

## Discussion

In this cross-sectional study consumption of unpasteurised soured or days-old livestock milk was associated with self-reported illness. Camel ownership, a proxy for contact with camels in the study's context [31], was also associated with a significant odds of self-reported illness, as was source of drinking water. These results are in line with previous work that has linked the potential transmission of zoonotic pathogens from camels and livestock to humans who are in close contact with them and the consumption of raw milk and other livestock products [3, 15, 32].

The sample size of 304 was the largest of the few studies focused on this study population, and its study design was informed by years of prior ethnographic work. Even so, this study was limited by reliance on self-report of disease and exposures. No biological samples were collected from either livestock or humans. Future studies that include sample collection and analysis are needed to better understand the aetiology of illness and to identify which zoonotic and food-borne pathogens are prevalent in this population.

Despite these limitations, this study fills important gaps in knowledge about common behavioural and dietary practices common among pastoralist and semi-pastoralist populations in Africa. Food preparation and consumption traditions among these populations may have important implications for the risk of zoonotic and foodborne diseases [8–10]. In particular, the consumption of raw, unpasteurised dairy products – including fresh milk, stored milk, soured milk and yoghurts – remains commonplace and consumption of days-old, unrefrigerated raw milk was associated with an increased odds of diarrhoea and vomiting in the study's population.

Food-borne zoonotic pathogens are major risks to population health among livestock herders like the ethnic Somalis studied here. For example, consumption of unpasteurised dairy products has been linked to an increased risk of zoonotic TB. Human TB cases from bovine TB infection are transmitted from infected animals through their bodily fluids, including milk, and likely contributes up to 10% of the global human tuberculosis burden yet remain understudied [14, 16, 33, 34]. Ethiopians and Ethnic Somalis have high rates of tuberculosis compared to other populations, even without a high prevalence of HIV/AIDS, and this could potentially be related to their exposure to bovine TB in livestock or raw livestock milk, although this possibility remains understudied [8–10, 14, 34]. There is a dearth of information about zoonotic TB in this setting [14, 34]. Nevertheless, a large study found that routine inspection of carcasses in Ethiopia detected 3.5% with lesions. However, a detailed inspection found it to actually be 10.2%, more than a three-fold difference [35].

Despite the risks of zoonoses, unpasteurised dairy products remain staple foods in pastoralists' and semi-pastoralists' diets in this part of the Horn of Africa, even during droughts and periods of population displacement. However, many Somalis in Ethiopia continue to experience chronic food insecurity and lack of access to veterinary health care for the livestock on which they depend. In addition, the study population has limited access to public works infrastructure and this is a well-documented risk factor for diarrhoeal diseases, zoonotic TB and other diseases. Future public health interventions should continue to invest in improving access to community-based veterinary care in places where pastoralists and semi-pastoralists settle, and begin to invest in the regulatory infrastructure necessary for the safe use of dairy products – especially in the rural and remote communities where livestock herding takes place.

## Data Availability

The data that support the findings of this study are available upon request from the corresponding author.
